# Clinical Characteristics, Neuroimaging Findings and Long-Term Sequelae in Children with Congenital Cytomegalovirus Infection: A Single Centre Study

**DOI:** 10.3390/pathogens14030280

**Published:** 2025-03-14

**Authors:** Ivana Valenčak, Lorna Stemberger Marić, Maja Vrdoljak Pažur, Snježana Židovec Lepej, Nenad Šuvak, Goran Tešović

**Affiliations:** 1 University Hospital for Infectious Disease “Dr. Fran Mihaljević”, 10000 Zagreb, Croatia; lmaric@bfm.hr (L.S.M.); mvrdoljak@bfm.hr (M.V.P.); szidovec@gmail.com (S.Ž.L.); gtesovic@bfm.hr (G.T.); 2 School of Medicine, University of Zagreb, 10000 Zagreb, Croatia; 3 School of Applied Mathematics and Infromatics, Josip Juraj Strossmayer University of Osijek, 31000 Osijek, Croatia; nsuvak@mathos.hr

**Keywords:** congenital cytomegalovirus infection, MRI findings, neurodevelopmental delay

## Abstract

Congenital cytomegalovirus infection is the most common congenital infection worldwide and an important cause of neurodevelopmental delay and sensorineural hearing loss. Neuroimaging represents the best prognostic marker in cCMV infection. The aim of this study was to establish the role of cranial ultrasound and brain magnetic resonance imaging in the development of long-term sequelae in symptomatic and asymptomatic children with cCMV infection. Of the 47 children enrolled in the study, 25 (53.1%) were classified as symptomatic at birth. In 27/47 patients, SNHL was diagnosed with a similar proportion among the symptomatic and asymptomatic at birth (51.8% and 48.1%, respectively; *p* = 1.0). Thirty case patients had available data on follow-up. Neurological sequelae were more frequently seen in patients with symptomatic cCMV, but only cerebral abnormalities seen on initial MRI results had a consequential link with the later development of motor (OR 17.5; 95% Cl: 2667, 114,846; *p* = 0.002) and speech disorders (OR 15; 95% Cl: 2477, 90,843; *p* = 0.02). Although not statistically significant, hearing deterioration was more frequent in children with abnormal MRI results (OR 5; 95% Cl: 0.846, 29,567; *p* = 0.121). Neuroimaging abnormalities, as identified through both cranial ultrasound (CrUS) and MRI, are critical prognostic indicators for long-term sequelae, applicable to both symptomatic and asymptomatic children.

## 1. Introduction

Congenital cytomegalovirus infection (cCMV) is the most common congenital viral infection worldwide with an incidence of 0.2–2.4% [1,2]. Although approximately 85–90% of infants with cCMV are asymptomatic at birth, cCMV is an important cause of neurodevelopmental delay and sensorineural hearing loss (SNHL) [3]. About 50–70% of symptomatic newborns develop permanent neurologic sequelae [2,4]. Up to 15% of infected infants that are asymptomatic at birth will develop sequelae as well [2]. In the absence of universal neonatal screening, cCMV is likely to remain undiagnosed in a significant proportion of those infected. Although the targeted screening of newborns who fail the neonatal hearing test would lower the proportion of undiagnosed children with cCMV, those with truly asymptomatic infection and risk for late-onset SNHL would remain undetected [5,6,7,8,9]. Brain involvement is the most serious consequence of cCMV, and neuroimaging is the best tool for predicting outcomes in affected children [10,11,12,13,14,15,16,17]. Antiviral treatment with ganciclovir/valganciclovir can prevent hearing deterioration and improve neurodevelopmental outcome [18,19].

In the present study, we analyzed the clinical characteristics at birth, neuroimaging findings and long-term sequelae in children with cCMV treated at the University Hospital for Infectious Diseases “Dr. Fran Mihaljević”, Zagreb, Croatia (UHID).

## 2. Materials and Methods

A retrospective analysis on the children treated for cCMV infection between January 2018 and December 2024 in the UHID was performed. cCMV infection was defined by the identification of viral DNA in urine samples within the first 21 days of life using polymerase chain reaction (PCR, COBAS^®^ AmpliPrep/COBAS^®^ TaqMan^®^ CMV Test, Roche Diagnostics, Mannheim, Germany).

Twenty-two children, in whom the infection was proven after 21 days of age, were also included in cCMV treatment, due to proven hearing impairment that could not be related to other causes or presented severe symptoms along with pathological findings compatible with cCMV on a brain MRI.

A symptomatic patient was defined as a newborn with CMV infection detected by PCR from a urine sample with at least one CMV infection-related sign or symptom present at birth: purpura/petechiae, jaundice, hepatosplenomegaly, unexplained neurological abnormality (hypotonia, lethargy, seizures), elevated liver enzymes (ALT > 40 IU/L), thrombocytopenia (platelet count < 150 × 10^9^/L), conjugated hyperbilirubinemia (direct bilirubin level > 2 mg/dL), abnormal ophthalmologic examination, microcephaly or life-threatening disease [20,21]. Infants who were small for gestational age (SGA) or had congenital SNHL without at least one of the aforementioned signs were considered asymptomatic [20].

Infants born between 37 and 40 weeks of gestation were defined as term, and infants born < 37 weeks as pre-term born. SGA and microcephaly were based on physicians’ examination at birth, defining SGA as a birth weight ≤ 10th percentile and microcephaly as a head circumference > 2 standard deviations (SD) below the mean for age and sex.

The abnormal cranial ultrasound (CrUS) and magnetic resonance imaging (MRI) findings associated with cCMV on the first evaluation included the following: calcifications (C), periventricular cysts (PVC), ventricular dilation (VD), subependymal pseudocysts (SEC), germinolytic cysts (GC), white matter abnormalities (WMA), cortical atrophy (CA), migration disorders (MD), cerebellar hypoplasia (CH) and lenticulostriatal vasculopathy (LSV). All CrUS procedures were performed by a neonatologist or pediatric infectious disease specialist while an experienced neuroradiologist evaluated all MRI findings.

Considering that this is a retrospective analysis of data, and at the time of treatment and diagnostic processing of the infected children, the Clinic for Infectious Diseases did not possess an MRI device, all children were referred for neuroradiological assessment (brain MRI) to another institution with which a collaboration agreement was established. If a child arrived with a pre-existing brain MRI, it was not repeated.

Audiological examination was conducted by an experienced pediatric audiologist and included the auditory steady-state response (ASSR), auditory brainstem response (ABR), behavioral audiometry (0.25–8 kHz) and tympanometry.

SNHL was categorized as mild (21–30 dBHL), moderate (31–60 dBHL), severe (61–90 dBHL) and profound (≥91 dBHL) for the click audiometry brainstem response, auditory steady-state response or pure-tone air conduction results, in the absence of middle ear disorders [22].

The ophthalmologic evaluation was conducted by an experienced pediatric ophthalmologist, and ophthalmologic abnormality associated with cCMV infection was defined if any of the following conditions were present: chorioretinitis, retinal hemorrhage, optic atrophy, strabismus, cataract.

Of the 47 children included in the study, 30 children were included in the final analysis, and 17 children were lost during the follow-up. Parents or legal guardians were contacted by phone for information on their child’s motor and speech development, improvement or worsening SNHL and vision impairment.

All variables analyzed were of either a categorical or discrete numerical type. For describing the data, we used the standard techniques, such as tables of frequencies and relative frequencies (proportions) as well as contingency tables for describing pairs of variables. Comparison between the categorical variables in the 2 groups was performed using Fisher’s exact test. Odds ratios (ORs) with 95% confidence intervals (CIs) were calculated for the presence of sequelae (hearing loss, motor and speech impairment) and the presence of abnormalities on MRI. Conclusions about the statistical significance of the differences of proportions were derived according to a binomial test on the 0.05 significance level. Statistical analysis was performed in the statistical software R, version 4.0.3.

## 3. Results

In total, forty-seven children were enrolled. The mean average age at admission was 60.3 days. Thirty-five patients were transferred from other pediatric wards and twelve were sent from primary care pediatricians to our center. All children sent from primary care pediatricians were older than 21 days of age at the time of admission. The main reasons for referring patients to our institution were deafness and/or pathological findings on CrUS or brain MRI results suggestive for cCMV. Among the 35 children previously treated in other hospitals (mostly in Neonatal Intensive Care Units), CMV viruria was detected in 10 (28.5%) after completing 21 days of life. All 10 patients had severe disease with clinical symptoms at birth and abnormal blood tests alongside pathological CrUS and brain MRI results. In eight (22.8%) patients, the diagnosis was established within the first three days of life, and in seventeen (48.5%), between the fourth and twenty-first days of life.

Of the 47 children, 25 (53.1%) were classified as symptomatic at birth. The most common cCMV-related sign present at birth was hypotonia (52%), while thrombocytopenia was the most common abnormal laboratory test (68%). All 14 patients born as SGA were previously classified with intrauterine growth restriction (IUGR) on fetal ultrasounds. Both SGA and IUGR had a statistically significant association with the development of symptomatic cCMV infection (*p* < 0.01 and *p* < 0.007, respectively). The main characteristics of the 25 patients with symptomatic cCMV at birth are summarized in Table 1.

In 34/47 patients, a brain MRI was performed at a median age of 53 days (range, 0–7 months). Among the patients who underwent CrUS, abnormal findings were registered in 34/45 (77.2%) while the brain MRI produced abnormal findings in 21/34 (61.7%). The results of the CrUS and MRI findings are listed in Table 2.

Of the 21 children with abnormal MRI findings, 20 had an abnormal initial CrUS. Of the seven patients with isolated LSV found on the CrUS, one had WMA while the rest had a normal brain MRI. WMA was the most common pathological MRI finding in symptomatic patients (11/17, 64.7%). Also, in only three patients, WMA was described on the CrUS followed by detection by MRI. In asymptomatic patients, the most common findings on the CrUS were LSV (10/21, 47.6%) and VD (5/21, 23.8%). In these patients, VD was the most common sign on the MRI (5/18, 27.7%), followed by WMA and SEC (in three and two patients, respectively). An abnormal CrUS was associated with a positive predictive value (PPV) of 95% in predicting an abnormal brain MRI.

All patients underwent audiological evaluation—the median age at first evaluation was 64 days (range, 17–130 days). In 27/47 patients, SNHL was diagnosed with a similar proportion among the symptomatic and asymptomatic at birth (51.8% and 48.1%, respectively; *p* = 1.0). A brain MRI was available in 12 children with SNHL, and 10 children with normal hearing. As indicated in Table 3, abnormal MRI findings were found both among children with and without SNHL (68.1% and 41.6%, respectively). Although not statistically significant, abnormal MRI results tended to be more frequent in children with SNHL (*p* = 0.13).

In three (6.3%) patients, an ocular abnormality had been diagnosed in neonatal age, including one with chorioretinitis, one with optic nerve atrophy and one with retinal hemorrhage. All three patients were symptomatic at birth and two of them had a pathological brain MRI result.

All of the 47 children received antiviral treatment for six months. Intravenous ganciclovir followed by oral valganciclovir was the most common option (27/47, 57.4%). Twenty patients (42.5%) were treated with valganciclovir only. Transient leukopenia was observed in three patients and anemia in two patients, but treatment was completed in all patients. 

Thirty case patients had available data on follow-up; the median age at the last evaluation was three years (range, one to seven years). Among these patients, 16/30 were classified as symptomatic at birth. Data regarding SNHL were collected from the 28 children available for follow-up. In 13/28 children, treatment was started within the first 28 days of life. In 15/28 children with SNHL, treatment was initiated after the neonatal period due to a delayed diagnosis. Hearing improvement was detected in 5/13 (38.4%) children that started therapy within 28 days of life, and deterioration was detected in 6/13 (46.1%). In children who started treatment after the 28th day of life, hearing improvement and deterioration were observed in 8/15 (53.3%) and 5/15 (33.3%), respectively. A “no difference in hearing” result in comparison with the first evaluation was found in four children (two in each group). Among twelve patients with normal hearing at first evaluation, only one developed delayed-onset SNHL. In three patients with normal hearing at first evaluation, their MRI results showed abnormalities, and one of them developed delayed onset SNHL.

One out of three patients with optic atrophy had deteriorated vision, while the other two had normal vision at follow-up at the age of three years.

Epilepsy was diagnosed in five (16.6%) patients and cerebral palsy in seven (23.3%) patients. Two of those patients were asymptomatic at birth, but all had a pathological MRI, with WMA as the most common finding (58.3%).

Motor and balance impairments were observed more frequently in patients with symptomatic cCMV (12/16, 75%) than in the asymptomatic group (4/14, 28.5%). Out of all the patients with motor impairments, an initial pathological MRI accounted for 14/16 (87.5%), with WMA as the most common finding (50%), followed by MD (35.7%). All patients with an asymptomatic infection at birth that later developed motor impairment had pathological findings on the initial MRI.

Speech and language disorders developed in both symptomatic and asymptomatic patients; nevertheless, they were more prevalent in patients that were symptomatic at birth (12/16, 75%). In patients with speech disorders, the MRI showed pathological findings in 15/18 (83.3%) patients, with WMA being the most frequent finding (53.3%).

Sequelae were more frequently seen in patients with symptomatic cCMV, but only cerebral abnormalities seen on the initial MRI had a consequential link with the later development of motor (OR 17.5; 95% Cl: 2667, 114,846; *p* = 0.002) and speech disorders (OR 15; 95% Cl: 2477, 90,843; *p* = 0.02). Although not statistically significant, hearing deterioration was more frequent in children with an abnormal MRI (OR 5; 95% Cl: 0.846, 29,567; *p* = 0.121) (Table 4).

## 4. Discussion

Multiple studies have explored prenatal and neonatal findings as potential biomarkers for the early identification of sequelae associated with cCMV infection. Regarding brain involvement, neuroimaging represents the most reliable indicator for CNS sequelae.

However, many of these studies are constrained by small sample sizes and a focus on predominantly symptomatic children, suggesting the necessity for additional validation of the results.

A combination of newborn screening and the screening of symptomatic infants, symptomatic infection, a high CMV viral load at birth and failed newborn hearing screening were identified as prognostic indicators for SNHL in the newborn period [23], while low birth weight, gestational age and prolonged viral shedding correlated with late-onset SNHL [24]. Moreover, studies that evaluated prenatal and neonatal cranial imaging, such as CrUS, CT and brain MRI, have shown inconsistent results, suggesting a potential link between imaging abnormalities, symptomatic disease and hearing loss [10,11,12,13,14,15,16,17,25,26,27]. Compared to CrUS, MRI provides important additional information regarding the detection of white matter lesions, migratory disorders and neocortical dysplasia [10,11,12,13,14,15,16,17,28]. Our results confirmed brain abnormalities that were detected on initial MRI scans as independent risk factors for motor and speech disorders. All of our patients with epilepsy, cerebral palsy and deteriorated hearing had an abnormal initial MRI. WMA and MD were the most common findings in MRI results in children with long-term sequelae. Several studies proposed a scoring system for the evaluation of neuroimaging in cCMV and to establish a prognosis [11,14,17]. Noyola’s scoring system was based on CT findings, so there is a concern about radiation exposure in children, and it has limitations if applied to MRI results [11]. Alarcon’s scoring system was based on MRI findings but was tested in a small number of patients [14]. Kwak et al. proposed a scoring system based on MRI findings in predicting a poor developmental outcome and epilepsy, but not SNHL [17]. All of the previous studies reported a correlation between abnormal findings CrUS, CT or MRI and the prediction of sequelae development in infants with cCMV [10,11,12,13,14,15,16,17]. However, there are limited data on children with a normal CrUS at birth and long-term sequelae. In the study by Capretti et al., among 40 enrolled children with cCMV, 34 had a normal CrUS with 3/34 having a brain abnormalities in the MRI and long-term sequelae [12]. In the Blázquez-Gamero et al. study, 3/7 children with a normal CrUS and an abnormal MRI manifested sequelae at 12 months of age [16]. In our study, one out of thirteen patients with a normal CrUS and an abnormal MRI developed sequelae during the follow-up. Regarding children without any symptoms at birth with an abnormal CrUS and/or MRI and long-term sequelae, there are only a few studies that have been performed on a small number of patients [13,16]. In the study by Ancora et al., SNHL developed in 3/37 neonates with a normal CrUS, whereas severe sequelae developed in one out of two neonates with an abnormal CrUS [12]. The study by Blázquez-Gamero et al., on 71 children with cCMV and available follow-up found that among children with a normal CrUS and MRI, thirteen out of twenty-two were asymptomatic and none developed sequelae, and nine out of twenty-two were symptomatic, of which two developed sequelae [16]. In our study, 5/14 (35.7%) asymptomatic children with available follow-up had an abnormal MRI and all of them developed sequelae (motor and speech disorders). All of these children also had an abnormal CrUS. Only one child who exhibited normal CrUS findings alongside abnormal MRI results developed long-term sequelae. Although we had a relatively small number of children included in the study, our results suggest that asymptomatic children with brain abnormalities in the MRI, especially WMA and MD, are at risk for long-term sequelae. No randomized clinical trials address this particular group of patients and more studies with a higher number of patients should be performed to clarify this debate. According to the European Congenital Infection Initiative (ECCI), MRI is recommended to infants who present clinical manifestations of CMV at birth, chorioretinitis or abnormalities detected by CrUS [29]. The question arises as to whether it is necessary to perform MRI to all children with cCMV. Our results suggest that CrUS is associated with a PPV of 95% in predicting an abnormal brain MRI. Brain ultrasound has proven to be a good screening method, and its ease of use (immediately at the child’s bedside, without the need for sedation) is an important advantage in using brain ultrasounds to detect the signs of brain damage associated with cCMV infection. Although ultrasound can depict most pathologies (ventriculomegaly, calcifications, cysts, LSV), its limitations are evident in detecting white matter lesions, migration disorders and cortical dysplasia, where a brain MRI is the primary diagnostic method. Given that these lesions are most closely associated with long-term consequences in terms of motor deficits, MRI is an important diagnostic method.

As was reported in other studies, children with symptoms at birth more frequently had abnormalities in the CrUS and MRI results, but we found six asymptomatic children with an abnormal MRI and CrUS. Following delivery, CrUS should be performed as a good screening tool for detecting brain involvement in all children with cCMV infection. Also, our findings suggested that in all asymptomatic children with an abnormal CrUS, a brain MRI should be performed. However, more studies should be performed before establishing brain MRI as an obligatory part of routine evaluation in asymptomatic children with cCMV and a normal CrUS.

In patients with SNHL, it is recommended to start antiviral treatment within the first 28 days of life in order to preserve/improve hearing [18,19,23]. As all our patients with SNHL received antiviral treatment for six months, we detected hearing improvement in the group of children in which treatment was started after 28 days of life. This implicates that some children with SNHL can benefit from antiviral therapy even if treatment started after 28 days of life. It is difficult to make strong conclusions from our results as to whether antiviral therapy is beneficial for SNHL improvement, even having started after the neonatal period, and in most cases, the last decision lies with clinicians. 

We acknowledge the limitations of our study. There was a higher proportion of symptomatic children and a higher occurrence of SNHL in asymptomatic children in our study, probably due to the cohorting of patients from other healthcare settings, as all of our patients either had a high probability of cCMV or had an already established diagnosis of cCMV. Although, in some cases where cCMV was diagnosed after the 21st day of life, we decide to include them in this study if they had clinical symptoms at birth or had abnormalities in the MRI results compatible with cCMV along with SNHL. We presumed that a late diagnosis was probably due to low cCMV awareness among physicians in Croatia. Another important limitation was the relatively small proportion of the children with available follow-up and retrospective analysis of the data. 

In conclusion, MRI abnormalities serve as independent risk factors for long-term sequelae in children with congenital cytomegalovirus (cCMV) infection. Asymptomatic congenitally infected children exhibiting abnormal brain MRI findings are at risk for developing long-term complications. Neuroimaging abnormalities, as identified through both cranial ultrasound (CrUS) and MRI, are critical prognostic indicators for long-term sequelae, applicable to both symptomatic and asymptomatic children. While CrUS is an effective screening modality for identifying brain involvement, it is recommended that children with an abnormal CrUS undergo a subsequent brain MRI for further evaluation. However, further investigation involving larger patient cohorts is necessary before establishing a definitive guideline for the routine use of brain MRI in asymptomatic congenitally infected newborns with normal hearing and CrUS findings.

## Figures and Tables

**Table 1:** Characteristics of the 47 children with cCMV included in this study and the distribution of clinical signs in 25 symptomatic children.

Characteristics	n (%)
Male	24 (51)
Female	23 (48.9)
Pre-term born infants	12 (25.5)
SGA	14 (29.7)
Symptomatic at birth	25 (53.1)
Distribution of clinical signs at birth in symptomatic patients	
Microcephaly	6 (24)
Conjugated hyperbilirubinemia	5 (20)
Petechiae/purpura	6 (24)
Hepatomegaly	10 (40)
Splenomegaly	8 (32)
Seizures	1 (4)
Hypotonia	13 (52)
SGA	14 (56)
Abnormal ophthalmologic examination	3 (12)
Thrombocytopenia	17 (68)
Elevated liver tests (ALT)	5 (20)
Hearing loss at birth	14 (56)

**Table 2:** Neuroimaging abnormalities of the 34 children with abnormal CrUS and 21 children with an abnormal brain MRI.

Neuroimaging Finding	Cranial Ultrasound n (%)	Brain MRI n (%)
Calcifications	6 (17.6)	6 (28.6)
Periventricular cysts	0	1 (4.8)
Ventricular dilation	12 (35.3)	9 (42.9)
Subependymal pseudocysts	6 (17.6)	3 (14.2)
Germinolytic cysts	0	
WM abnormalities	3 (8.8)	14 (66.7)
Cortical atrophy	2 (5.9)	2 (9.5)
Migration disorders	0	5 (23.8)
Cerebellar hypoplasia	0	1 (4.8)
Lenticulostriatal vasculopathy	21 (61.8)	0
Hemorrhage to germinative matrix (hemosiderin)	0	2 (9.5)

**Table 3:** Association between MRI findings and SNHL at birth in 47 children with cCMV.

	Symptomatic	Asymptomatic
Total number of children (%)	25	22
Number (%) of children with SNHL at birth	14 (56)	13 (59)
Unilateral hearing loss	8 (57.1)	5 (38.5)
Bilateral hearing loss	6 (42.8)	8 (61.5)
Number of children with SNHL and available MRI	12 (85.7)	10 (76.9)
Number (%) of children with abnormal MRI findings and SNHL	11 (50)	4 (18.2)

**Table 4:** Association between abnormal MRI findings and the presence of sequelae in 30 children with available follow-up and outcomes.

Sequelae	Incidence of Sequelae Positive/Total Examined (%) Normal MRI (n = 12)	Incidence of Sequelae Positive/Total Examined (%) Abnormal MRI (n = 18)	Odds Ratio (95% Cl)	*p* Value	Relative Risk
Motor and balance disorders	2/12 (16.7)	14/18 (77.8)	17.5 (2667, 114,846)	0.002	5714 CI (1499, 21,781)
Seizures	0/12	5/18 (27.8)	NE	0.065	NE
Cerebral palsy	0/12	7/18 (38.9)	NE	0.024	NE
Speech disorders	3/12 (25)	15/18 (83.3)	15 (2477, 90,843)	0.002	4.5 CI (1523, 13,296)
Hearing deterioration	2/12 (16.7)	9/18 (50)	5 (0.846, 29,567)	0.121	2895 CI (0.770, 10,882)
Ocular abnormalities	0/12	1/18 (5.5)	NE	1.0	NE

NE, could not be estimated.

## Data Availability

All data generated or analyzed during this study are included in the published article.

## Abbreviations

The following abbreviations are used in this manuscript: cCMV, congenital cytomegalovirus infection; SNHL, sensorineural hearing loss; UHID, University Hospital for Infectious Diseases “Dr. Fran Mihaljević”; MRI, magnetic resonance imaging; PCR, polymerase chain reaction; SGA, small for gestational age; SD, standard deviation; CrUS, cranial ultrasound; C, calcifications; PVC, periventricular cysts; VD, ventricular dilation; SEC subependymal pseudocysts; GC germinolytic cysts; WMA, white matter abnormalities; CA, cortical atrophy; MD, migration disorders; CH, cerebellar hypoplasia; LSV, lenticulostriatal vasculopathy; IUGR, intrauterine growth restriction; ECCI, European Congenital Infection Initiative.
